# The tongue coating microbiome is perturbed in atrial fibrillation and partly normalized after catheter ablation

**DOI:** 10.3389/fmicb.2025.1508089

**Published:** 2025-04-30

**Authors:** Ling Wang, Na Li, Yuchen Zheng, Qiong Huang, Guangying Cui, Xiaoshuai Cheng, Yu He, Yifei Niu, Yumei Sun, Xiaoming Wang, Hong Luo, Pengfei Liu, Junjie Tan, Bingsen Huang, Li Li, Peiyao Ma, Dandan Li, Yanyan Li, Jing Li, Zujiang Yu, Zhigang Ren, Yiqiang Yuan

**Affiliations:** ^1^Department of Clinical Laboratory, Henan Provincial Chest Hospital, Zhengzhou University, Zhengzhou, Henan, China; ^2^Department of Infectious Diseases, State Key Laboratory of Antiviral Drugs, Pingyuan Laboratory, The First Affiliated Hospital of Zhengzhou University, Zhengzhou, China; ^3^Affiliated Henan Cardiovascular Hospital, Southern Medical University (Zhengzhou Seventh People's Hospital), Zhengzhou, Henan, China; ^4^Department of Cardiovascular Medicine, Henan Provincial Chest Hospital, Zhengzhou University, Zhengzhou, Henan, China; ^5^Department of General Surgery, Guangshan County People’s Hospital, Xinyang, Henan, China; ^6^Department of Cardiovascular Medicine, Guangshan County People’s Hospital, Xinyang, Henan, China; ^7^Department of Clinical Laboratory, Guangshan County People’s Hospital, Xinyang, Henan, China

**Keywords:** tongue coating microbiome, atrial fibrillation, catheter ablation, biomarkers, diagnosis

## Abstract

**Background:**

There is accumulating evidence linking the microbiome and cardiovascular diseases. Nevertheless, no existing studies have been conducted on atrial fibrillation (AF) and the oral microbiome.

**Materials and methods:**

We collected and sequenced 245 AF tongue-coating samples and 26 AF samples after catheter ablation from Zhengzhou and Guangshan, China. We characterized tongue coating microbiome, constructed microbial classifiers in the discovery cohort, and verified their diagnostic potential in a cross-regional cohort.

**Results:**

Tongue coating microbial richness and diversity were significantly increased in the AF group compared to the control group, indicating increased bacterial colonization. The classifiers based on four optimal tongue coating microbial markers achieved good diagnostic efficiency in AF cohorts, with area under the curve (AUC) of 99.10 and 98.62% in the discovery and validation cohorts, respectively, and 97.97% in the cross-regional cohort. Paroxysmal AF and persistent AF shared similar taxonomic features, but some specific differential bacteria acted in the AF progression. Moreover, the outcomes revealed that catheter ablation contributed to rehabilitating oral bacterial disorders.

**Conclusion:**

This was the first cross-sectional and longitudinal research of oral microbiome in AF patients and the alternations after catheter ablation, which offers promising new perspectives for AF clinical diagnosis and management.

## Background

Atrial fibrillation (AF) places an enormous burden on healthcare systems worldwide. Considerable research endeavors are being devoted to obtaining detailed information about the underlying mechanisms and effective treatment (Hindricks et al., 2021). AF itself induces progressive functional and structural changes in the atrial myocardium that facilitate the long-term perpetuation of the arrhythmia (Martins et al., 2014; Quintanilla et al., 2019). Based on the duration of episodes, AF can be classified into paroxysmal AF (PAF, which lasts <7 days) and persistent AF (psAF, which lasts ≥7 days), whereas the added predictive value of biomarkers is presently not well defined (Hindricks et al., 2021). Catheter ablation (CA) is currently the first-line treatment strategy for rhythm control due to its effectiveness at sustaining sinus rhythm (Asad et al., 2019), but the recurrence rate is high and it is prone to complications. The pathogenesis and progression of AF have not been thoroughly elaborated, so it is pivotal to explore the underlying mechanism to prevent and manage it.

The oral microbiome or oralome is second only to the gut microbiome in abundance and diversity, with 772 known species found in the oral cavity (Verma et al., 2018). The past decade has witnessed substantial growth in the body of research on the oral microbiome and its potential role in promoting cardiovascular diseases, comprising atherosclerotic cardiovascular diseases (Sen et al., 2018), heart failure (Molinsky et al., 2022), infective endocarditis (Drangsholt, 1998), and rheumatic heart disease (McDonald et al., 2004). Dysbiosis of the oral microbiome contributes to cardiovascular diseases through biofilm formation, endothelial dysfunction, molecular mimicry, platelet aggregation, direct arterial invasion, and systemic inflammation (Tonelli et al., 2023). Nevertheless, a link between oral microbiome and AF has not yet been established. At present, although some studies have demonstrated an effective role of biomarkers in AF risk assessment (Hijazi et al., 2017), there is uncertainty over the exact time point of biomarker assessment, optimal cut-offs, and the effect on management decision-making based on changes in biomarker levels over time. The dynamic nature of the microbiome changing with the host disease state makes it a potential biomarker for predicting the risk of AF development and progression.

Considering the emerging correlation between the human oral microbiome and cardiovascular diseases, we sought to explore whether oral microbial dysbiosis was related to the AF course and the possibility of oral microbial biomarkers for AF diagnosis. Therefore, we recruited a cohort of AF patients (including PAF and psAF) and those undergoing catheter ablation from Henan Province, China, and collected tongue coating swabs early in the admission period and at 6 months post-procedure. Our novel results uniquely characterized both horizontally and vertically how oral microbial ecological dysregulation might be engaged in the disease dynamics of AF, as well as established a diagnostic model for AF that enabled cross-regional validation.

## Materials and methods

### Study profile

**Figure 1:**
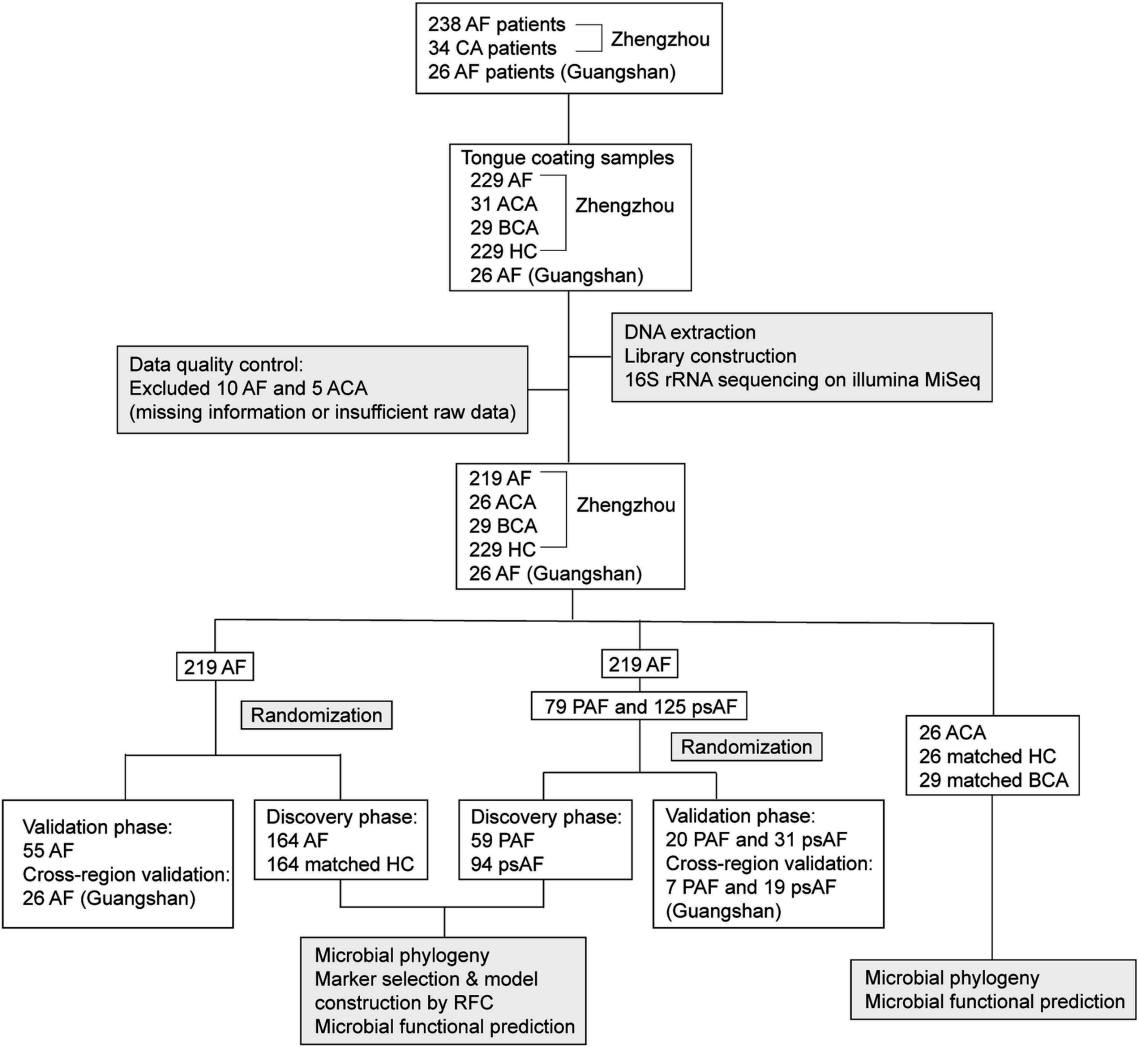
Study design and flow diagram. A total of 544 tongue coating samples from Henan Provincial Chest Hospital and Guangshan County People’s Hospital were collected. After rigorous inclusion and exclusion criteria, 529 samples were included for further analysis, including 219 AF, 26 ACA, 29 paired BCA, 229 HC samples from Zhengzhou and 26 AF samples from Guangshan. Tongue coating samples were sequenced using 16S rRNA MiSeq to characterize the microbiome and construct predictive or diagnostic model. AF, atrial fibrillation; ACA, 6 months after catheter ablation; BCA, before catheter ablation; HC, healthy control; PAF, paroxysmal AF; psAF, persistent AF; RFC, random forest classifier; rRNA, ribosomal RNA.

This research was performed based on the prospective specimen collection and retrospective blinded evaluation design principles (Pepe et al., 2008). We recruited 264 AF patients who were hospitalized in Henan Provincial Chest Hospital and Guangshan County People’s Hospital from May 1, 2021 to April 30, 2023 (Figure 1). All enrolled patients received standard treatment according to “Current knowledge and management of atrial fibrillation: consensus of Chinese experts 2021” (Chinese Society of Pacing and Electrophysiology, Chinese Society of Arrhythmias, Atrial Fibrillation Center Union of China, 2022). After rigorous inclusion and exclusion criteria, 529 samples were included for 16S rRNA MiSeq sequencing. Among them, samples from AF patients treated with catheter ablation were collected before the procedure and 6 months after the procedure. Clinical data and participants’ demographics were collected and analyzed (Table 1). Supplementary material showed inclusion, exclusion, and detailed diagnostic criteria of the participants. The study conformed to the principles of the Declaration of Helsinki. The research protocol was approved by the ethics committee of Henan Provincial Chest Hospital (2021-05-05). All participants signed informed consent.

**Table 1:** Clinical characteristics of participants in the oral discovery cohort.

Clinical indexes	AF patients	Healthy controls	*p* value
	(n = 164)	(n = 164)	
Age (years)	62.11 ± 14.19	63.29 ± 11.99	0.415
Sex (female/male)	62/102	71/93	0.311
AF types (PAF/psAF)	77/87	-	-
Glucose	5.51 (5.02, 6.65)	5.31 (5.06, 5.72)	0.012
Hemoglobin A1c	6.15 ± 1.14	5.76 ± 0.47	0.002
Total cholesterol	4.15 (3.54, 5.01)	4.65 (4.13, 5.14)	<0.001
Triglyceride	1.18 (0.89, 1.70)	1.47 (0.99, 2.04)	0.004
Low-density lipoprotein	2.28 ± 0.79	2.84 ± 0.76	<0.001
Creatine Kinase	72.00 (47.00, 103.00)	87.50 (70.50, 106.25)	0.348
Creatine kinase MB	1.60 (0.78, 4.61)	-	-
Lactate dehydrogenase	171 (146, 202)	202.00 (181.50, 242.50)	0.033
Aspartate aminotransferase	25.00 (19.65, 32.90)	21.00 (17.75, 26.00)	<0.001
Alanine aminotransferase	24.05 (15.90, 36.20)	19.00 (14.00, 27.00)	0.004
Gamma-glutamyl transferase	28.75 (20.55, 49.50)	21.00 (14.25, 35.00)	<0.001
Total protein	66.50 (61.93, 70.90)	74.6 (72.08, 77.03)	<0.001
Albumin	40.70 (36.88, 43.50)	48.20 (46.58, 49.43)	<0.001
Globulin	26.30 (23.50, 29.45)	26.45 (23.80, 29.03)	0.849
Total bilirubin	15.44 ± 7.78	12.17 ± 5.51	<0.001
Direct bilirubin	3.60 (2.50, 5.40)	4.75 (3.78, 6.05)	<0.001
Indirect bilirubin	10.60 (7.43, 15.00)	6.80 (4.80, 9.50)	<0.001
Creatinine	71.86 ± 26.12	71.85 ± 15.09	0.994
Uric acid	333.73 ± 138.42	343.96 ± 90.37	0.436
Blood urea nitrogen	6.67 ± 6.07	4.91 ± 1.18	<0.001
White blood cells	6.12 ± 1.80	6.14 ± 1.49	0.889
Red blood cells	4.51 ± 0.78	4.87 ± 0.76	<0.001
Blood platelet	187.00 (144.00, 224.00)	227.50 (192.00, 257.75)	<0.001
C-reactive protein	1.75 (0.51, 3.78)	4.40 (3.09, 4.82)	<0.001

Continuous variables are presented as mean ± standard deviation or median (interquartile range). Categorical variables are presented as percentages. Differences between subjects with AF (*n* = 164) and healthy controls (*n* = 164) were compared by using Student’s t-test for normal continuous variables, the Wilcoxon rank-sum test for non-normal continuous variables and the χ2 test or Fisher’s exact test for categorical variables. Statistical significance was defined by *p* < 0.05 (two-tailed). ARB, angiotensin receptor antagonist; ACEI, angiotensin converting enzyme inhibitor; NYHA, New York Heart Association.

### Tongue-coating sample collection and DNA extraction

Before taking tongue-coating samples, participants used sterile water to rinse their mouths twice. The posterior middle to anterior middle area of the tongue coating was scraped by a professional operator with a pharyngeal swab. The swab was immediately placed into a cryotube, the virus was inactivated at 56°C for 30 min and then the sample was transferred to the freezer at −80°C.

The sample was suspended in 790 μL of sterile lysis buffer (4 M guanidine thiocyanate; 10% N-lauroyl sarcosine; 5% N-lauroyl sarcosine-0.1 M phosphate buffer [pH 8.0]) in 2 mL screw-cap tube containing 1 g glass beads (0.1 mm BioSpec Products, Inc., United States). This mixture was vortexed vigorously and then incubated at 70°C for 1 h. After incubation by bead beating for 10 min at maximum speed. DNA was extracted by following the manufacturer’s instructions for bacterial DNA extraction using The E.Z.N.A. ® Stool DNA Kit (Omega Bio-tek, Inc., GA), which excepted lysis steps and stored at −20°C for further analysis.

### PCR amplification and MiSeq sequencing

The primers F1 and R2 (5′-CCTACGGGNGGCWGCAG-3′ and 5′-GACTACHVGGGTATCTAATCC-3′) correspond to positions 341 to 805 in the *Escherichia coli* 16S rRNA gene were used to amplify the V3-V4 region of each sample by PCR. PCR reactions were run in a T100™ Thermal Cycler PCR system (Bio-Rad Laboratories, Inc., United States) using the following program: 3 min of denaturation at 95°C followed by 21 cycles of 0.5 min at 94°C (denaturation), 0.5 min for annealing at 58°C, and 0.5 min at 72°C (elongation), with a final extension at 72°C for 5 min.

The amplicons from different samples were purified by Hieff NGS® DNA Selection Beads (YeasenBiotechCo., Ltd., China). The products were indexed and mixed at equal ratios for sequencing by Shanghai Mobio Biomedical Technology Co., Ltd. using the Miseq platform (Illumina Inc., United States) according to the manufacturer’s instructions. Raw Illumina read data for all samples were deposited in the National Center for Biotechnology Information (PRJNA1061363).

### Operational taxonomy unit clustering and taxonomy annotation

The non-repetitive sequences were extracted in the optimized sequences, which facilitated the reduction of redundant computations in the intermediate process of the analysis.^1^ Single sequences without repeats were removed.^2^ Operational taxonomy unit (OTU) clustering of non-repeated sequences (excluding single sequences) was performed according to 97% similarity, and chimeras were removed in the clustering process to obtain the OTU representative sequences. All the optimized sequences were mapped to the representative sequences of OTUs, and the sequences with 97% or more similarity to the representative sequences of OTUs were selected to generate a table of OTUs. The OTU representative sequences with a 97% similarity level were taxonomically analyzed at each taxonomic level: phylum, class, order, family, genus, and species were counted for the community composition of each sample. The comparative databases were 16S bacterial and archaeal ribosomal databases Silva^2^ (Release 138).^3^

### Microbial diversity and taxonomic analysis

Alpha diversity metrics (Ace estimator, Chao 1 estimator, Shannon-Wiener diversity index, and Simpson diversity index) were assessed by using Mothur v1.42.1. Both Bray-Curtis and weighted and unweighted UniFrac dissimilarity were calculated in QIIME. PCoA, NMDS plots, Adonis, and ANOSIM, which were applied to test for statistical significance between the groups, were generated in the R (version 3.6.0) package vegan 2.5–7. The linear discriminant analysis (LDA) effect size (LEfSe)^4^ was used to detect taxa with differential abundance among groups.

### Identification of the OTU biomarkers and construction of probability of disease

The discovery OTU frequency profile, validation frequency profile, and independent diagnosis frequency profile were generated by mapping reads from the discovery cohort, validation cohort, and independent diagnosis cohort against these represented sequences, respectively. Then, we used the Wilcoxon rank-sum test to determine the significance (*p* < 0.05), and OTU biomarkers in the tongue coating microbiomes were selected for further analysis. We constructed a diagnostic model through fivefold cross-validation on a random forest model and evaluated the probability of disease (POD) index and receiver operating characteristic curve. The process was performed as we described in Supplementary material.

### Bioinformatics and statistical analysis

PICRUSt2 v2.4.1^5^ was used to predict functional abundances based on 16S rRNA gene sequences. Differences between the two groups were compared using Student’s t-test for normal continuous variables, the Wilcoxon rank-sum test for non-normal continuous variables, and the χ2 test or Fisher’s exact test for categorical variables. Differences among groups were assessed by using one-way analysis of variance for normal continuous variables and the Kruskal-Wallis test for non-normal continuous variables. Differences with a *p*-value < 0.05 (two-sided) were considered statistically significant. We used a multiple comparison false positive correction. To control for the false positive rate associated with multiple comparisons, we corrected the *p*-values of all comparisons for false discovery rate (FDR). Specifically, we used the Benjamini-Hochberg process for FDR correction. This method first sorted all p-values according to the original significance level and then calculated a threshold for each p-value in the sorted order to control the FDR at a predetermined level. Statistical analyses were performed using IBM SPSS Statistics 26 for Windows (IBM Corp., Armonk, New York).

## Results

### Study design and flow diagram

After excluding, 529 samples were included for further analysis (Figure 1), including 219 AF, 26 ACA, 29 paired BCA, 229 HC samples from Zhengzhou, and 26 AF samples from Guangshan. Tongue-coating samples from AF and HC were randomly divided into the discovery phase and validation phase in a ratio of 3:1. In the discovery phase, we characterized the tongue coating microbiome in 164 AF and 164 matched HC samples, identified the key tongue coating microbial markers, and constructed AF classifiers by a fivefold cross-validation random forest model. In the validation phase, we verified the diagnostic efficacy of the AF classifier based on the tongue coating microbiome in 55 AF samples. Furthermore, 26 tongue-coating samples from Guangshan were applied to validate the cross-regional diagnostic efficacy of the AF classifier. In addition, we divided AF into two groups (79 PAF and 125 psAF samples), and comparatively characterized the oralome of different AF types. Moreover, we also examined the dynamics of the tongue coating microbiota in AF patients before catheter ablation (BCA) (29 samples) and 6 months after catheter ablation (ACA) (26 samples).

### Baseline characteristics of the study cohort

In the discovery cohort, the clinical characteristics of AF and HC were shown in Supplementary Table S1. Age and sex were matched between the AF and HC groups (*p* > 0.05). In the AF groups, compared with HC, total cholesterol (TC) (*p* < 0.001), triglyceride (TG) (*p* < 0.05), low-density lipoprotein (LDL) (*p* < 0.001), lactate dehydrogenase (LDH) (*p* < 0.05), total protein (TP) (*p* < 0.001), albumin (ALB) (*p* < 0.001), direct bilirubin (DBIL) (*p* < 0.001), red blood cells (RBC) (*p* < 0.001), blood platelet (PLT) (*p* < 0.001), and C-reactive protein (CRP) (*p* < 0.001) were decreased, while glucose (GLU) (*p* < 0.05), hemoglobin A1c (HbA1c) (*p* < 0.05), aspartate aminotransferase (AST) (*p* < 0.001), alanine aminotransferase (ALT) (*p* < 0.05), gamma-glutamyl transferase (GGT) (p < 0.001), total bilirubin (TBIL) (*p* < 0.001), indirect bilirubin (IBIL) (*p* < 0.001), and blood urea nitrogen (UA) (*p* < 0.001) was increased. We performed a correlation analysis of bacteria in the tongue coating microbiota with data on medication use (anticoagulants, antiarrhythmics, lipid-lowering drugs, antihypertensives, antiplatelets, and amiodarone) and medical history (cerebral infarction, hypertension, and type 2 diabetes mellitus) using Masslin2 (Supplementary Figure S1). We excluded these bacteria associated with clinical indicators from the differential bacteria between AF and healthy individuals.

### Microbial dysbiosis in the tongue coat of AF patients

**Figure 2:**
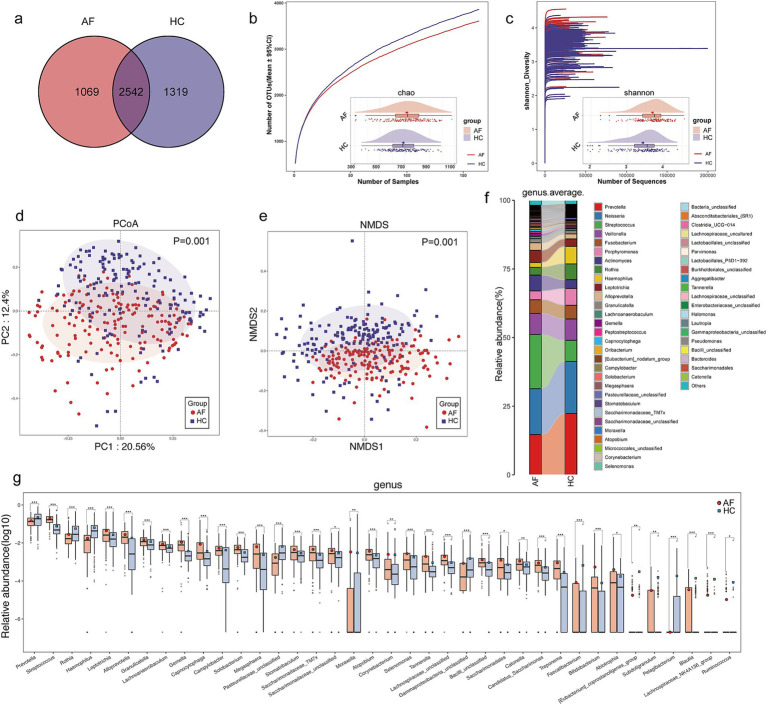
Microbial dysbiosis in the tongue coat of AF patients. **(a)** A Venn diagram displayed that 2,542 of 4,930 OTUs were common to both AF and HC groups, while 1,069 OTUs were unique to the AF. **(b)** Rarefaction analysis between the number of samples and the number of OTUs. As the number of samples increased, the number of OTUs approached saturation. Compared with the HC, the number of OTUs in AF was decreased. As estimated by the Chao index, tongue coating microbial richness was significantly increased in AF compared with HC. **(c)** Shannon-Wiener curves of samples showed that as the number of sequences increased, the Shannon diversity approached saturation. As estimated by the Shannon index, tongue coating microbial diversity was significantly increased in AF compared with HC. The PCoA **(d)** and NMDS **(e)** (Bray-Curtis distance) analysis showed the tongue coating microbial taxonomic composition was conspicuously different between the two groups. The ratio of the variance contribution was shown. **(f)** Average compositions and relative abundance of the bacterial community in both groups at the genus level. **(g)** At the genus level, 37 microbial features were enriched in AF, while 15 microbial features were depleted. AF, atrial fibrillation; HC, healthy control; OTUs, operational taxonomy units; PCoA, principal coordinate analysis; NMDS, nonmetric multidimensional scaling. ^*^p < 0.05, ^**^p < 0.01, ^***^p < 0.001.

The Venn diagram displayed that 2,542 of 4,930 OTUs were common to both AF and HC groups, while 1,069 OTUs were unique to the AF (Figure 2a). The tongue coating microbial diversity of AF and controls was evaluated through the Shannon index and Chao 1 for alpha diversity, principal coordinates analysis (PCoA) and nonmetric multidimensional scaling (NMDS) for beta diversity. Rarefaction analysis of the discovery cohort showed that OTU diversity and richness in each group approached saturation (Figures 2b,c). As estimated by the Shannon index (*p* < 0.001) and Chao 1 index (*p* < 0.05), tongue coating microbial diversity and richness were increased in the AF versus HC (Figures 2b,c; Supplementary Table S2). Both PCoA and NMDS plots calculated by Bray-Curtis distance revealed that tongue coating microbiome separated AF and controls into two strikingly distinct groups (*p* = 0.001, ANOSIM) (Figures 2d,e; Supplementary Figures S2a,b), indicating that tongue coating microbiome dysbiosis occurred in AF.

Further analysis of AF and HC composition and alterations uncovered that genera *Prevotella*, *Neisseria*, *Streptococcus*, *Veillonella*, and *Fusobacterium* were the five leading genera in both groups (Figure 2f; Supplementary Table S3), and the phylum *Firmicutes*, *Bacteroidota*, *Proteobacteria*, *Actinobacteriota*, and *Fusobacteriota* were the most five abundant phyla, accounted for over 95% of sequences on average (Supplementary Figure S2c; Supplementary Table S4). At the genus level, 37 microbial features were enriched in AF, including *Streptococcus*, *Actinomyces*, and *Leptotrichia*, while 15 microbial features were depleted, including *Prevotella*, *Rothia*, and *Haemophilus* (Figure 2g; Supplementary Table S5). At the phylum level, the phylum *Firmicutes*, *Actinobacteriota*, *Fusobacteriota*, *Patescibacteria*, *Campilobacterota*, and *Spirochaetota* were increased, and the phylum *Bacteroidota* and *Proteobacteria* were diminished in the AF compared with controls (Supplementary Figure S2d; Supplementary Table S6). We further performed linear discriminant analysis (LDA) effect size (LEfSe) analysis and selected the most representative genera closely correlated with AF in accordance with LDA (LDA > 2.5, *p* < 0.05) (Supplementary Figure S2e; Supplementary Table S7). These findings indicated that unique tongue coating microbiome profiles were present in AF groups.

### A non-invasive diagnostic model for AF based on the tongue coating microbiome

**Figure 3:**
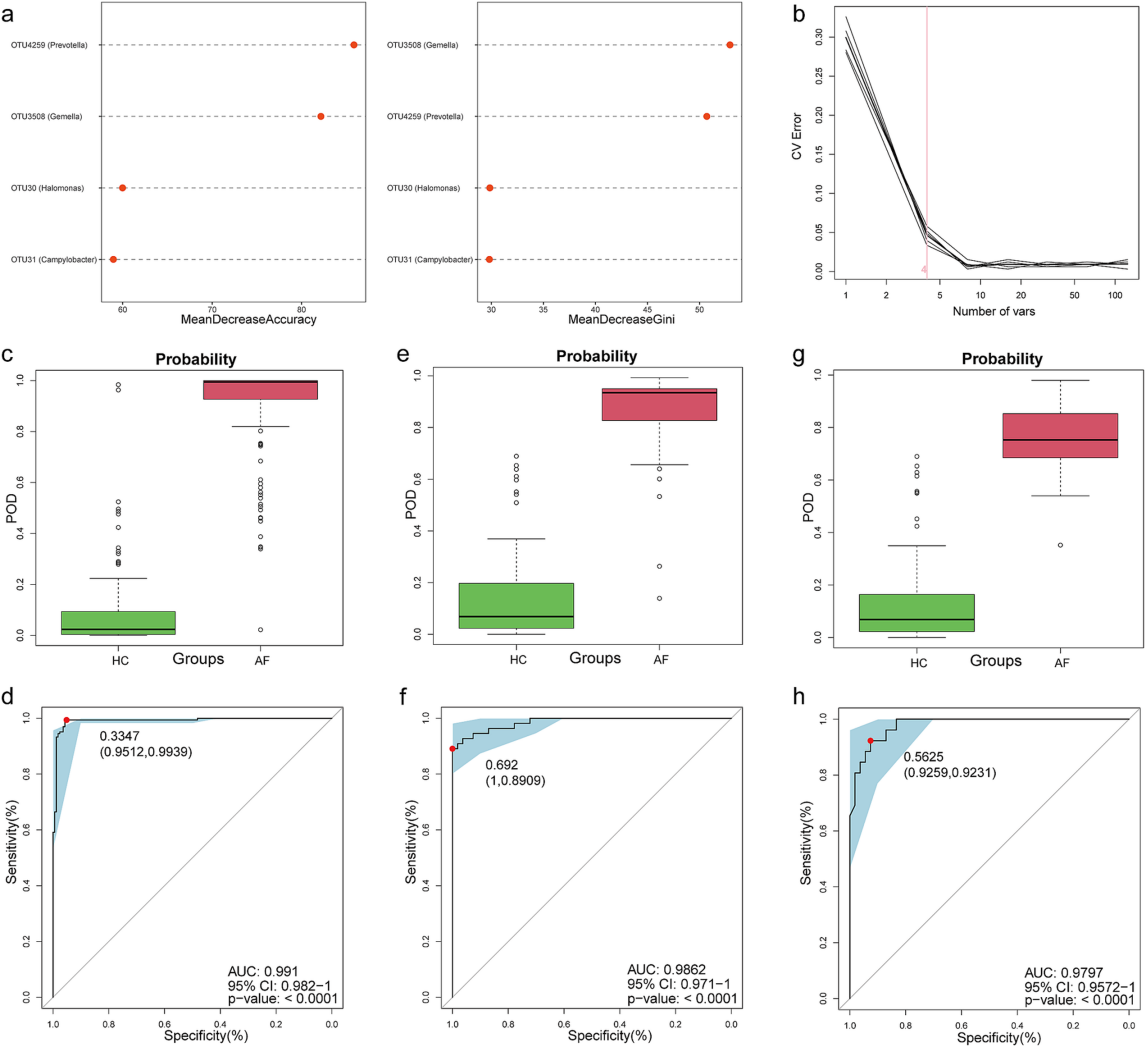
Identification of a tongue coating microbial classifier for AF. **(a)** Four microbial markers were selected as the best markers set by random forest model. **(b)** Importance distribution map of the selected microbial markers in the model. The POD value was significantly increased in AF compared with HC, and achieved good diagnostic efficacy in the discovery cohort **(c,d)**, the validation cohort **(e,f)**, and the independent cohort **(g,h)**. AF, atrial fibrillation; HC, healthy control; POD, probability of disease; AUC, area under the curve. Centerline, median; box limits, upper and lower quartiles; circle or square symbol, mean; error bars, 95% CI.

To assess the diagnostic value of tongue coating microbial markers for AF, we constructed a random forest classifier model between 164 AF and 164 matched HC. Initially, 4 OTUs that could accurately identify differences between both groups were selected as the optimal marker set through fivefold cross-validation in the random forest model (Figures 3a,b). Then, we calculated the POD index of the discovery cohort by using a 4-OTU set (Supplementary Table S8). The POD index was markedly higher in AF than in HC (Figure 3c), and it reached an AUC of 99.10% (95% CI 98.20 to 100%, *p* < 0.0001) (Figure 3d). These data indicated that oral microbial markers could specifically identify patients with AF from HC.

Meanwhile, 55 AF in the validation cohort were used to verify the diagnostic efficacy of microbial biomarkers. The POD index (Figure 3e; Supplementary Table S9) was significantly higher in AF, with an AUC value of 98.62% (95% CI 97.10% to 100%) between both groups (*p* < 0.0001) (Figure 3f). Additionally, we further collected 26 AF tongue-coating samples from Guangshan, which served as an independent diagnostic. The POD index (Figure 3g; Supplementary Table S10) was higher in 26 Guangshan AF versus HC, with an AUC value of 97.97% (95% CI 95.72 to 100%) between both groups (*p* < 0.0001) (Figure 3h). These results suggested that this classifier based on the oral microbiome for AF has powerful diagnostic efficacy.

### Tongue coating microbial characterization among PAF and psAF

**Figure 4:**
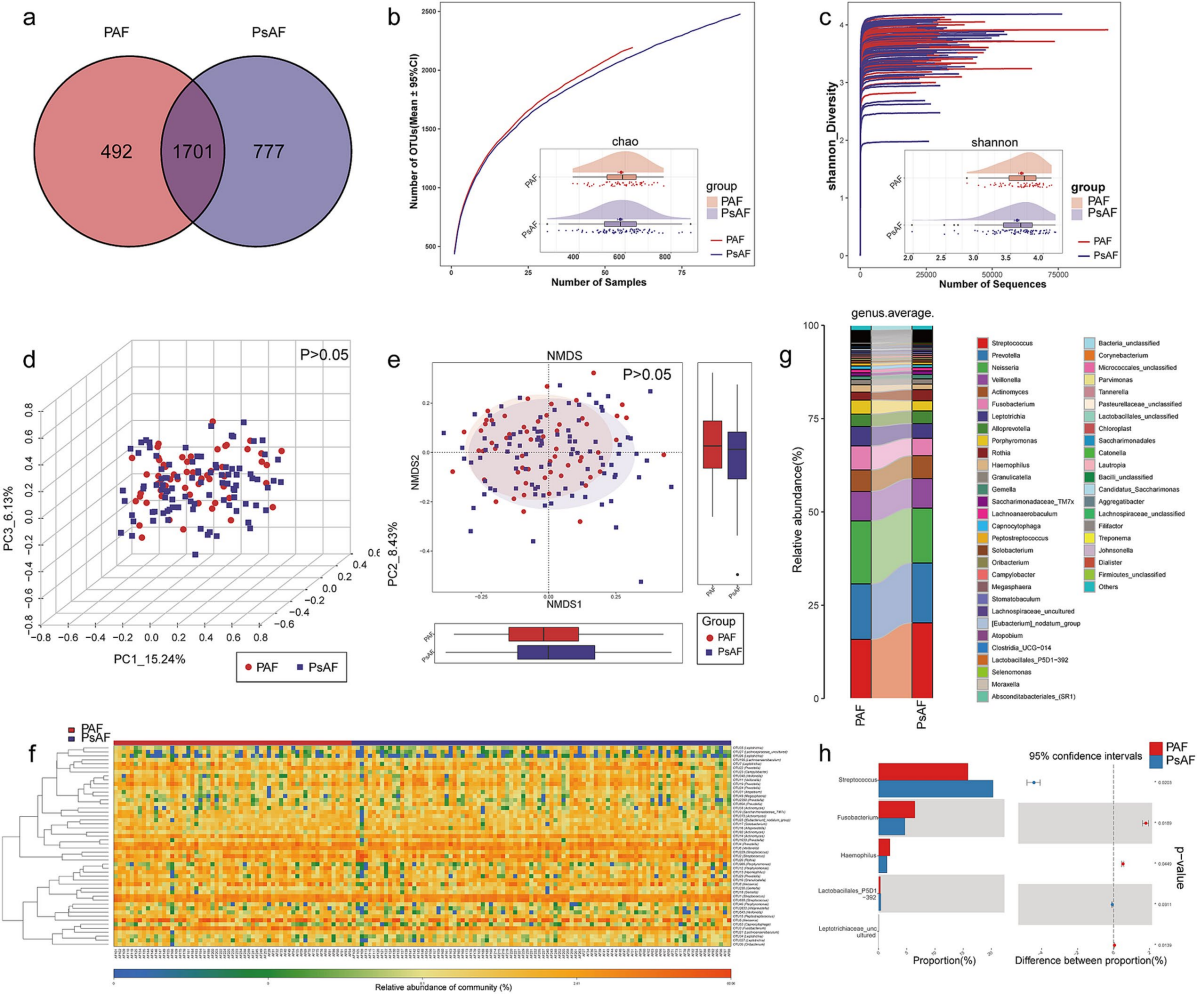
Tongue coating microbial characterization among PAF and psAF. **(a)** A Venn diagram displayed that 1,701 of 2,970 OTUs were shared between PAF and psAF, while only 492 and 777 OTUs were unique to the PAF and psAF, respectively. **(b)** Rarefaction analysis between the number of samples and the number of OTUs. As the number of samples increased, the number of OTUs approached saturation. As estimated by the Chao index, the tongue coating microbial richness of PAF and psAF was similar. **(c)** Shannon-Wiener curves of samples showed that as the number of sequences increased, the Shannon diversity approached saturation. As estimated by the Shannon index, tongue coating microbial diversity was similar in PAF and psAF. The PCoA **(d)** and NMDS **(e)** (Bray-Curtis distance) showed that there was no significant difference in the tongue coating microbiome distribution between PAF and psAF. The ratio of the variance contribution was shown. **(f)** Heatmap for the relative abundances of differential OTUs for each sample in the PAF and psAF groups. **(g)** Average compositions and relative abundance of the bacterial community in the PAF and psAF groups at the genus level. **(h)** Compared with PAF, two genera were increased, while three genera were depleted in psAF. PAF, paroxysmal AF; psAF, persistent AF; OTUs, operational taxonomy units; PCoA, principal coordinate analysis; NMDS, nonmetric multidimensional scaling. ^*^p < 0.05, ^**^p < 0.01, ^***^p < 0.001.

According to AF history and manifestation of cardiogram, AF patients were classified as PAF (*n* = 79) and psAF (*n* = 125). A Venn diagram showed that 1,701 of 2,970 OTUs were shared between PAF and psAF, while only 492 and 777 OTUs were unique to the PAF and psAF, respectively (Figure 4a). The tongue coating microbial diversity and richness of PAF and psAF were similar (*p* > 0.05) (Figures 4b,c; Supplementary Table S11). Although there was no significant difference, Shannon and Chao 1 index were decreased in psAF patients. The heatmap, PCoA, and NMDS analysis failed to distinguish different AF patients (*p* > 0.05, ANOSIM) (Figures 4d–f; Supplementary Figure S2f; Supplementary Table S12). These findings indicated that AF patients possess similar microbial features in tongue coating diversity, regardless of whether they manifested clinically as PAF or psAF.

The average composition and relative abundance of the tongue coating microbiome at the genus and phylum levels were displayed in Figure 4g, Supplementary Figure S2g, and Supplementary Tables S13, S14. Although PAF and psAF shared similar taxonomic characteristics, there were also some specific differential bacteria. Compared with PAF, two genera including *Streptococcus* and *Lactobacillales*_P5D1–392 were increased, while three genera including *Fusobacterium*, *Haemophilus*, and *Leptotrichiaceae*_uncultured were depleted in psAF (all *p* < 0.05) (Figure 4h; Supplementary Table S15). These results demonstrated a further increase in *Streptococcus* and *Lactobacillales*_P5D1–392, while a further decrease in *Haemophilus* during the AF progression. Different analyses at the phylum level are presented in Supplementary Figure S2h and Supplementary Table S16. Overall, these results indicated that the oral microbial abundances and composition in PAF were basically consistent with those in psAF, but some specific AF-related bacteria changed more significantly in psAF.

### Alterations in the tongue coating microbiome in AF patients 6 months after catheter ablation

**Figure 5:**
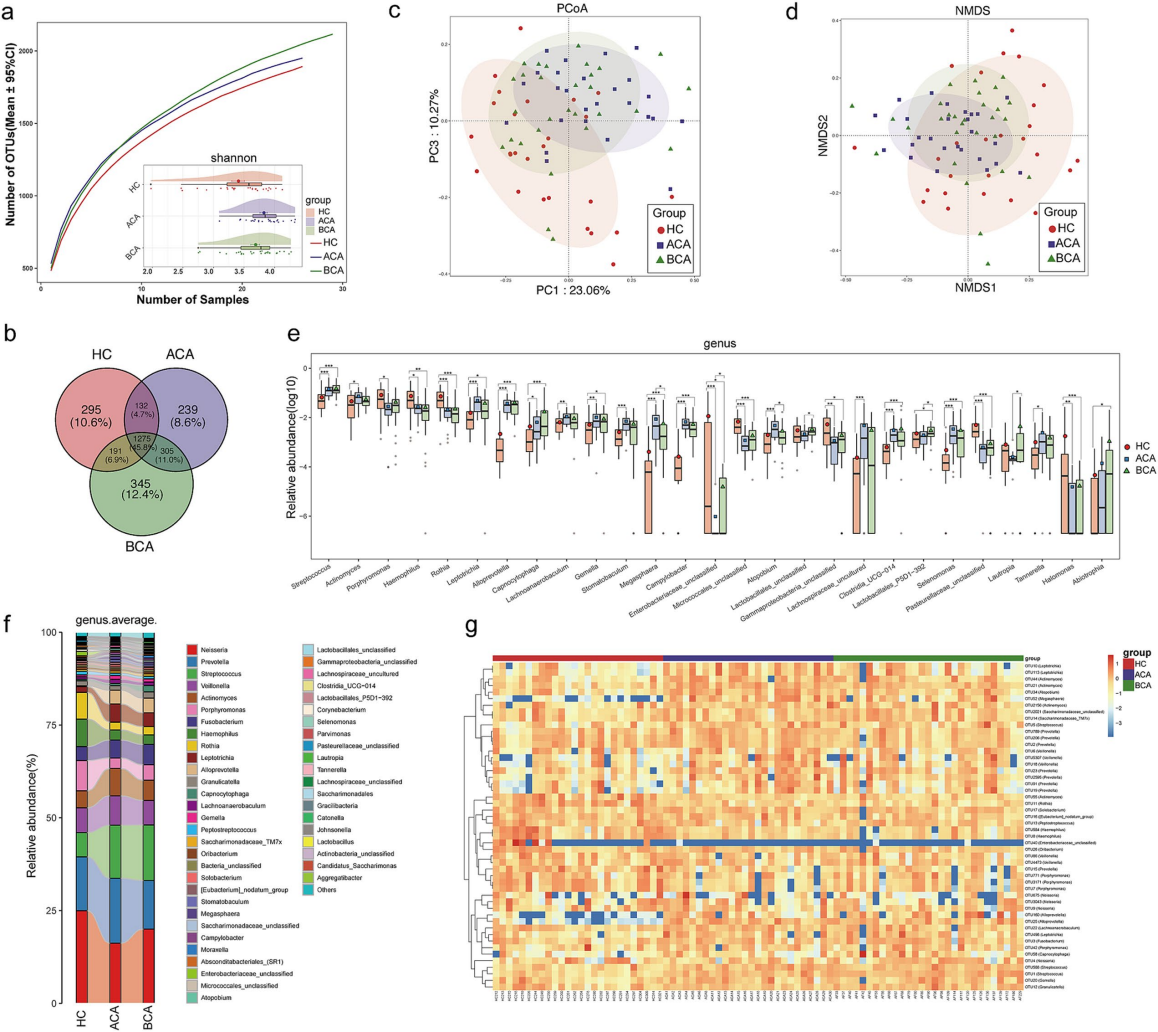
Alterations in tongue coating microbiome in AF patients 6 months after catheter ablation. **(a)** Rarefaction analysis between the number of samples and the number of OTUs. As the number of samples increased, the number of OTUs approached saturation. Compared with the HCs, the number of OTUs in ACA and BCA was increased. As estimated by the Shannon index, the tongue coating microbial diversity of ACA and BCA was similar but significantly increased compared with that of the HC. **(b)** A Venn diagram revealed that 1,407 of 1,951 OTUs in ACA were shared with HC, and 1,580 OTUs were shared with BCA. PCoA **(c)** and NMDS **(d)** (Bray-Curtis distance) displayed that the tongue coating microbial distribution in ACA was comparable to that of BCA but distinct from that of HC. The ratio of the variance contribution was shown. **(e)** The abundance of 15 genera gradually decreased after catheter ablation and 12 genera gradually increased. **(f)** Average compositions and relative abundance of the bacterial community in the three groups at the genus level. **(g)** Heatmap for the relative abundances of differential OTUs for each sample in the three groups. HC, healthy control; ACA, 6 months after catheter ablation; BCA, before catheter ablation; OTUs, operational taxonomy units; PCoA, principal coordinate analysis; NMDS, nonmetric multidimensional scaling. ^*^p < 0.05, ^**^p < 0.01, ^***^p < 0.001.

We explored and compared the tongue coating microbiome characteristics in 26 ACA, 29 matched BCA, and matched HC. The tongue coating microbial diversity in the ACA was similar to that in the BCA but increased compared with that in the HC (*p* < 0.05) (Figure 5a; Supplementary Table S17). Microbial richness did not differ significantly among the three groups (Supplementary Table S17). A Venn diagram revealed that 1,407 of 1,951 OTUs in ACA were shared with HC, and 1,580 OTUs were shared with BCA (Figure 5b). PCoA and NMDS displayed that the tongue coating microbial distribution in ACA was comparable to that of BCA (*p* > 0.05, Adonis) but distinct from that of HC (*p* < 0.05, Adonis) (Figures 5c,d; Supplementary Figure S2i).

The average composition and relative abundance of the tongue coating microbiome for three groups at the genus and phylum levels were displayed in Figure 5f, Supplementary Figure S2k, and Supplementary Tables S18, S19. The most dominant microbes in BCA and HC were *Neisseria* but were *Prevotella* in ACA. The differential microbes among the three groups displayed that the microbial characterization of ACA was significantly different from HC but similar to BCA (Figure 5g; Supplementary Table S20). We further performed differential analysis at the genus and phylum levels (Figure 5e; Supplementary Figure S2j; Supplementary Tables S21, S22). The outcomes demonstrated a total of 288 different genera in the three groups, of which the abundance of 15 genera gradually decreased after catheter ablation, including *Lactobacillales*_P5D1–392, *Lautropia*, and *Lactobacillales*_unclassified, while 12 genera gradually increased. We conducted LEfSe analysis and selected the most representative genera among three groups on LDA (LDA > 2.5, *p* < 0.05) (Supplementary Figure S2l; Supplementary Table S23). These data indicated that dynamic alterations in the tongue coating microbiome might reflect or contribute to post-ablation pathophysiological processes in AF patients.

### Crucial tongue coating microbial predicted functions correlated with AF progression

**Figure 6:**
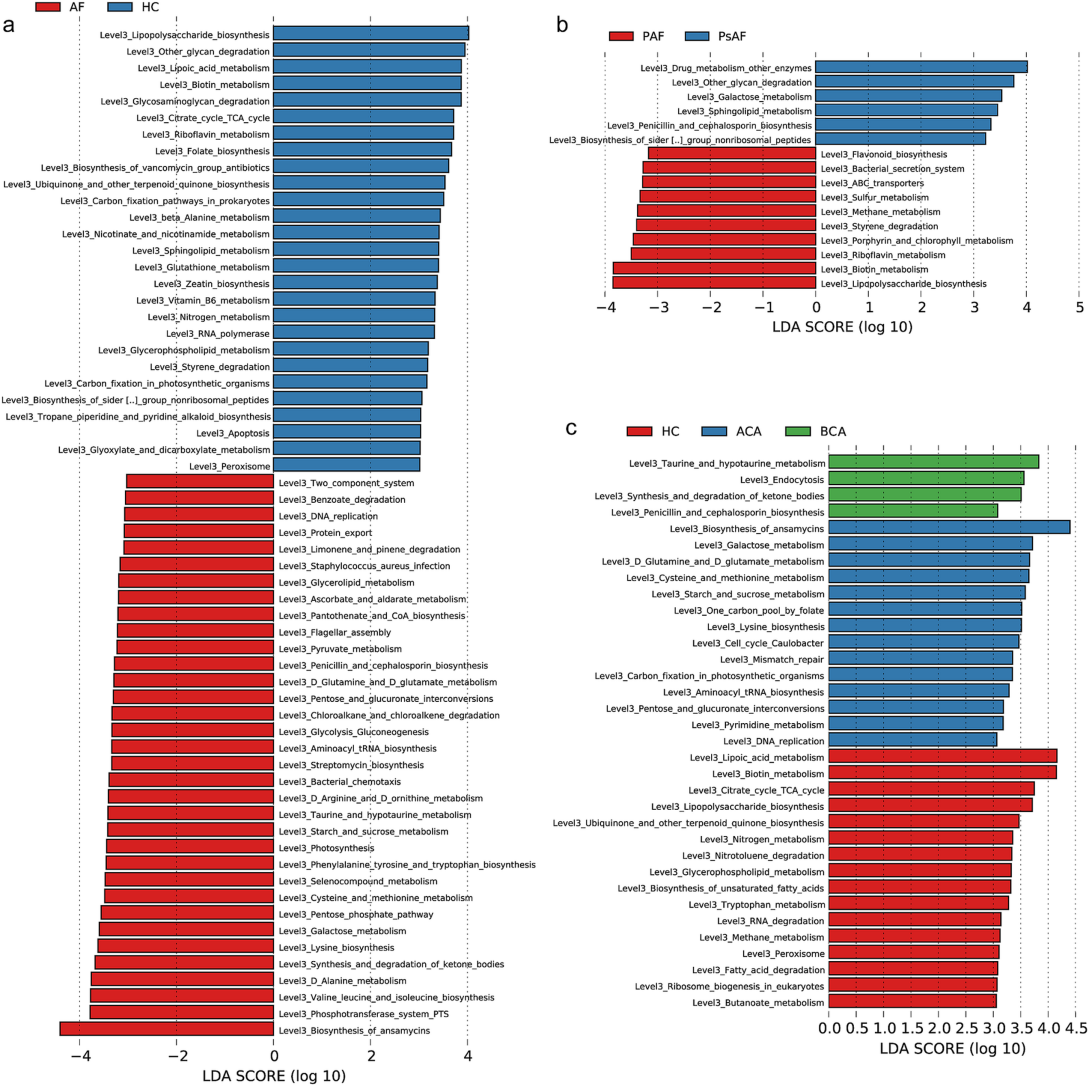
Crucial tongue coating microbial predicted functions correlated with AF progression. **(a)** Compared with HC, 34 functional modules increased significantly, while 27 functions decreased significantly in AF (LDA > 3, *p* < 0.05). **(b)** Compared with PAF, 6 functional modules increased significantly, while 10 functions decreased significantly in AF (LDA > 3, *p* < 0.05). **(c)** Based on the LDA selection, 4 functions were significantly increased in BCA, 14 functions were remarkedly raised in ACA, while 16 functions were notably increased in HC (LDA > 3, *p* < 0.05). AF, atrial fibrillation; HC, healthy control; PAF, paroxysmal AF; psAF, persistent AF; ACA, 6 months after catheter ablation; BCA, before catheter ablation; LDA, linear discriminant analysis.

The enriched pathways in which the tongue coating microbiome might affect the progression of AF were identified in the KEGG database. Compared with HC, 34 functional modules, including biosynthesis of ansamycins, synthesis and degradation of ketone bodies, and galactose metabolism increased significantly, while 27 functions, including lipopolysaccharide biosynthesis, lipoic acid metabolism, and citrate cycle decreased significantly in AF (LDA > 3, *p* < 0.05) (Figure 6a; Supplementary Table S24). Compared with PAF, 6 functional modules, including drug metabolism (other enzymes), galactose metabolism, and sphingolipid metabolism increased significantly, while 10 functions, including lipopolysaccharide biosynthesis, biotin metabolism, and riboflavin metabolism decreased significantly in AF (LDA > 3, *p* < 0.05) (Figure 6b; Supplementary Table S25). The results displayed that 14 pathways were enriched in the ACA group, such as biosynthesis of ansamycins, galactose metabolism, as well as glutamine and D glutamate metabolism (LDA > 3, *p* < 0.05) (Figure 6c; Supplementary Table S26). Compared with HC, many pathways were depleted in the ACA and BCA groups, such as lipoic acid metabolism, biosynthesis of unsaturated fatty acids, and butanoate metabolism. These outcomes implied dysbiosis of functional patterns in AF progression.

### Associations between the tongue coating microbiome and clinical indicators

**Figure 7:**
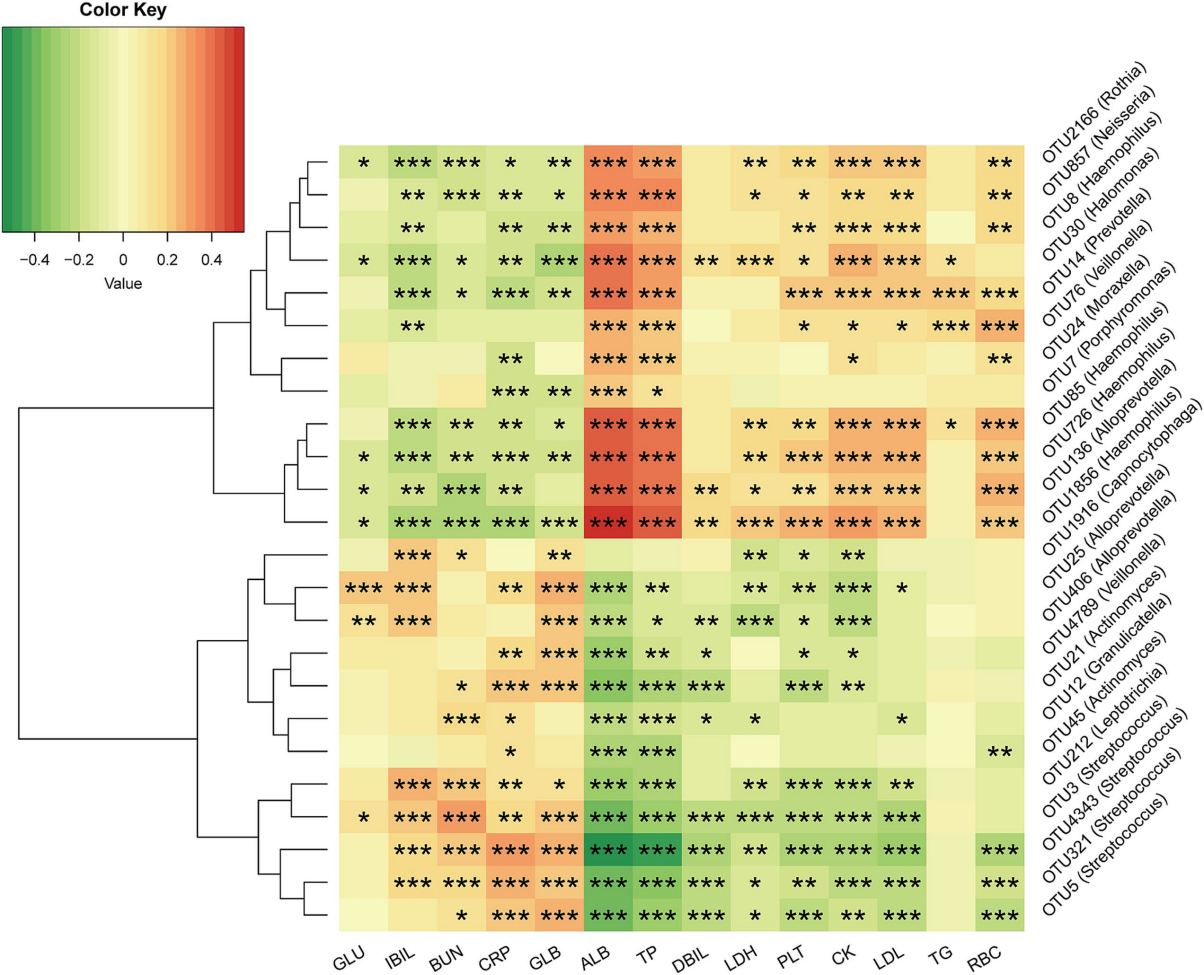
Associations between the tongue coating microbiome and clinical indicators. Spearman correlations of significantly altered tongue coating microbiome with significantly altered clinical indicators in AF and HC. Red color represents the positive correlation, and green color represents the negative correlation. GLU, glucose; IBIL, indirect bilirubin; BUN, blood urea nitrogen; CRP, C-reactive protein; GLB, globulin; ALB, albumin; TP, total protein; DBIL, direct bilirubin; LDH, lactate dehydrogenase; PLT, blood platelet; CK, Creatine kinase; LDL, low-density lipoprotein; TG, triglyceride; RBC, red blood cells; OTU, operational taxonomy unit. ^*^p < 0.05, ^**^p < 0.01, ^***^p < 0.001.

The correlation between tongue coating microbiome and clinical indicators was identified in AF patients and controls through Spearman correlation analysis (p < 0.05 and absolute rho > 0.2) (Figure 7; Supplementary Table S27). We found that 2 clinical indicators (GLU and TG) were correlated with no more than 10 OTUs, while 12 clinical indicators (IBIL, BUN, CRP, GLB, ALB, TP, DBIL LDH, PLT, CK, LDL, and RBC) were related to more than 10 OTUs. CK were positively correlated with 11 OTUs, including OTU1856 (*Haemophilus*) and OTU14 (*Prevotella*) (*p* < 0.05), and negatively correlated with 10 OTUs, including OTU4343 (*Streptococcus*) and OTU21 (*Actinomyces*) (*p* < 0.05). CRP was positively correlated with 10 OTUs, including OTU4343 (*Streptococcus*) and OTU21 (*Actinomyces*) (*p* < 0.05), and negatively correlated with 11 OTUs, including OTU1856 (*Haemophilus*) and OTU14 (*Prevotella*) (*p* < 0.05). This explained the interaction of tongue coating microbiome, cardiac enzymes, liver and kidney function, as well as routine blood tests, pathways that might be involved in disease progression.

## Discussion

Our study first identified compositional and functional alterations in the oral microbiome linked to AF horizontally and longitudinally. We discovered specific microbial markers (e.g., *Streptococcus*, *Prevotella*) and constructed a diagnostic model that yielded favorable diagnostic results in two large cohorts from different regions. We also revealed dynamic variations of the oral microbiota in different AF types and after catheter ablation therapy.

The oral microbiome as a mediator of systemic inflammation and metabolic dysfunction is increasingly recognized in cardiovascular diseases (Tonelli et al., 2023). The richness and diversity of the oral microbiome has been evaluated in multiple diseases, particularly in cardiovascular diseases (Sun et al., 2023). We observed significantly higher tongue coating bacterial richness and diversity index in the AF group compared to the control group, indicating overgrowth of a variety of harmful bacteria and fewer commensal or beneficial genera. Investigations of gut microbial characterization in AF patients uncovered consistent alterations in gut bacterial and viral diversity with our results (Zuo et al., 2019; Zuo et al., 2022b). As with previous research on gut microbiota (Zuo et al., 2020; Huang et al., 2022), tongue coating microbial richness and diversity did not alter significantly in different AF types (PAF and psAF) as well as in the short term after catheter ablation. This phenomenon might be related to oral-gut microbial transmission, which has been linked to various disease conditions (Kunath et al., 2024). Orally originated genera such as *Streptococcus* might co-colonize ectopically in the gut to affect AF. Moreover, proton-pump inhibitors, which altered gut microbiota by promoting oral-originated *Streptococcus* translocation into gut (Xiao et al., 2024), warranted further study in AF treatment.

Oral microorganisms formed polymicrobial binding where bacteria were stimulated to generate glycoproteins and polysaccharides to build ecological niches termed biofilms (Verma et al., 2018). We considered that the oralome was correlated with AF through biofilms, which were linked to low-level immune activation and systemic inflammation (Sintim and Gürsoy, 2016). Our findings indicated an elevated prevalence of opportunistic pathogens in AF patients, including *Streptococcus*, *Actinomyces*, and *Leptotrichia* as well as a decreased prevalence of commensal bacteria, including *Prevotella*, *Rothia*, and *Haemophilus*. *Streptococcus sanguinis* could express platelet aggregation-associated protein 43 (Herzberg, 1996) and serotype k of *Streptococcus mutans* could express collagen-binding adhesins (Nakano et al., 2010), which contributed to AF through modulation of platelet aggregation. Antibodies against periodontal microorganisms (including *Actinomyces naeslundii*) might reduce AF mortality (Qi et al., 2020). Oral commensal bacteria might affect structural remodeling of the atria through secreted proteins in atherosclerotic plaques associated with bacterial pathogenesis, virulence enhancement, and host immune modulation and regulation (Chhibber-Goel et al., 2016). These findings position oral biofilms as a reservoir of pro-arrhythmic mediators, bridging oral ecology to systemic inflammation.

The progression from PAF to psAF was marked by a striking increase in *Streptococcus* and *Lactobacillus*_P5D1–392 associated with inflammatory burden in autoimmune diseases (Guo et al., 2023). We hypothesized that chronic atrial stretch in psAF created a pro-inflammatory milieu favoring acid-tolerant *Streptococcus* species. Intriguingly, catheter ablation partially restored *Haemophilus* levels, potentially via improved cardiac output enhancing mucosal oxygenation, a critical factor for symbiotic *Haemophilus* growth (Xu et al., 2023). Furthermore, the post-ablation resurgence of *Actinomyces* underscored the need for adjunctive antimicrobial strategies to prevent AF recurrence. Their interventions could be an effective strategy to decelerate the progression and prevent the recurrence of AF, which deserved further research.

The oralome also could promote cardiovascular diseases through the production of metabolites, which acted as immunostimulators or immunomodulators that translocated into the circulation from the oral cavity. Increased levels of the ketone body *β*-hydroxybutyrate promoted histone acetylation of the Sirt7 promoter and activated Sirt7 transcription, leading to cardiomyocyte apoptosis and cardiac fibrosis (Xu et al., 2021), suggesting that microbial metabolites served as epigenetic and metabolic regulators of arrhythmogenesis. Citrate synthase played a protective role in regulating AF development (Teng et al., 2022). Several ceramide and sphingomyelin species were associated with incident AF, and these associations differ based on the fatty acid (Jensen et al., 2020). Moreover, lipopolysaccharide-induced NLRP3-inflammasome and pro-inflammatory macrophages diminished the atrial effective refractory period, elicited atrial electrical remodeling, and enhanced AF inducibility (Sun et al., 2016; Zhang et al., 2022b). However, the metabolic function of lipopolysaccharides was found to be decreased in AF patients. We hypothesized that this might be related to the renin- and angiotensin-converting enzyme inhibitors, angiotensin receptor blockers, and statins taken by AF patients, which could reduce vascular oxidative stress and have vasoprotective effects (Förstermann and Sessa, 2012).

Oral microbiome testing was clinically validated for systemic lupus erythematosus (Guo et al., 2023), demonstrating that microbiological diagnostics were feasible in mainstream medicine. Our 4 OTUs biomarker panel had very high diagnostic accuracy (AUC > 97%) and cross-regional validity, making it an ideal screening tool for AF in primary care. Unlike electrocardiograms, which required arrhythmia episodes to be active during testing, oral microbiome analysis provided a non-invasive, pre-symptomatic window of detection-a critical advantage for early intervention in at-risk populations such as those with high blood pressure or diabetes (Hindricks et al., 2021). Moreover, longitudinal microbiome surveillance for AF patients could identify individuals with persistent *Streptococcus*/*Lactobacillus*_P5D1–392 enrichment, suggesting a higher risk of recurrence after ablation and guiding personalized antimicrobial prophylaxis. However, standardizing sampling protocols and determining diagnostic thresholds in different populations remained a challenge.

Biomarkers could be a potential candidate for specific targeted therapies (Dang et al., 2021; Tran et al., 2023). Targeting oral ecological dysregulation and its metabolic functions, which represented a frontier in precision cardiology, were potential novel therapies for the prevention and treatment of AF. Lipoic acid derivatives could reduce inflammation and oxidative stress, and thus reverse cardiac fibrosis (Fukunaga et al., 2012; Sardu et al., 2017). Oral administration of *Bacteroides fragilis* significantly attenuated inflammatory response by increasing Treg cells, thereby preventing atrial structural remodeling and inhibiting AF promotion in D-galactose-induced aging rats (Zhang et al., 2022a). Moreover, short-chain fatty acid derived from dietary fiber fermentation by oral and gut commensals alleviated AF development via G-protein-coupled receptor 43/NLRP3 signaling (Zuo et al., 2022a). Notably, intensive periodontal therapy has been documented to lower HbA1c levels in patients with type 2 diabetes mellitus (D'Aiuto et al., 2018). HbA1c served as a reliable risk factor for all-cause mortality and cardiovascular mortality (Cavero-Redondo et al., 2017), indicating the importance of routine oral health assessment and periodontitis treatment for the effective management of AF.

We endeavored to elucidate the alterations and potential contributions of oral microbiome in AF, but this work still has several shortcomings. First, although this study identified a link between oral dysbiosis and AF, the exact causal relationship has not been clarified, and further interventional and pathomechanistic studies are warranted. Second, we have only investigated oral microbial genomics, and subsequent work will further explore metagenomic, metabolomics, transcriptomics, and proteomics by collecting serum and fecal specimens in larger cohorts. Third, the oral microbiome refers to the assembly of microbiota, including bacteria, fungi, viruses, protists and archaea, microbial structural elements, and their internal and external structural elements (Berg et al., 2020). However, this study explored only the oral bacteria, while additional research will be conducted in the future to investigate other microorganisms and their correlations with bacteria. Fourth, there remains an urgent requirement for the discovery of new and effective treatments for oral dysbiosis, as well as for robust randomized clinical trials of existing therapies. Finally, smoking and alcohol consumption, exercise, dietary and proton-pump inhibitors information were not collected and corrected in this study, and their effects on oralome require further characterization. Validation of the diagnostic model based on a larger sample size and basic research on specific bacteria and metabolites will be conducted in the future.

## Conclusion

This was the first cross-sectional and longitudinal research of oral microbiome in AF patients, intending to substantiate the bi-directional relationship between AF and oral microbiome. Our findings uniquely described how oral microbial dysbiosis was involved in the AF onset and progression while establishing a diagnostic model that enabled cross-regional validation. Although more mechanistic studies and clinical validation are needed, these findings provided a novel perspective on the pathology, diagnosis, and treatment of AF.

## Data Availability

The datasets generated and/or analyzed during the current study are available in the National Center for Biotechnology Information (accession number: PRJNA1061363). Correspondence and requests for materials should be addressed to YY.

## Footnotes

^1^ http://drive5.com/usearch/manual/dereplication.html

^2^ http://drive5.com/usearch/manual/singletons.html

^3^ http://www.arb-silva.de

^4^ https://github.com/SegataLab/lefse

^5^ https://github.com/picrust/picrust2/

## Supplementary material

SUPPLEMENTARY FIGURE S1 Heatmap of tongue flora associated with medication use and medical history data at genus level. T2DM, type 2 diabetes mellitus; CI, cerebral infarction.

SUPPLEMENTARY FIGURE S2 **(a)** Heatmap based on Bray-Curtis distances reflect the degree of variation in species abundance distributions among samples. **(b)** Box plots of the results of ANOSIM’s analysis of similarity between AF and HC groups and comparisons of differences within and between groups. **(c)** Average compositions and relative abundance of the microbial community in AF and HC groups at the phylum level. **(d)** 6 phyla were remarkedly increased, while 2 phyla were remarkedly reduced in AF versus controls. **(e)** Linear discriminant analysis (LDA) effect size in oral microbiome between AF and HC groups at genus level (LDA > 2.5, *p* < 0.05). **(f)** Box plots of the results of ANOSIM’s analysis of similarity between PAF and psAF groups and comparisons of differences within and between groups. **(g)** Average compositions and relative abundance of the microbial community in PAF and psAF groups at the phylum level. **(h)** 1 phylum was remarkedly increased, while 1 phylum was remarkedly reduced in psAF versus PAF. **(i)** Beta diversity calculated by Adonis among ACA, BCA, and HC groups. **(j)** The differential microbes among ACA, BCA, and HC groups at the phylum level. **(k)** Average compositions and relative abundance of the microbial community in ACA, BCA, and HC groups at the phylum level. **(l)** LDA effect size in oral microbiome among ACA, BCA, and HC groups at genus level (LDA > 2.5, *p* < 0.05). AF, atrial fibrillation; HC, healthy control; PAF, paroxysmal AF; psAF, persistent AF; ACA, 6 months after catheter ablation; BCA, before catheter ablation. ^*^*p* < 0.05, ^**^*p* < 0.01, ^***^*p* < 0.001.
