# Supplementary figures and images for: The tongue coating microbiome is perturbed in atrial fibrillation and partly normalized after catheter ablation
Source: Front Microbiol. 2025 Apr 30;16:1508089. doi: 10.3389/fmicb.2025.1508089 (PMC12075123; doi:10.3389/fmicb.2025.1508089)

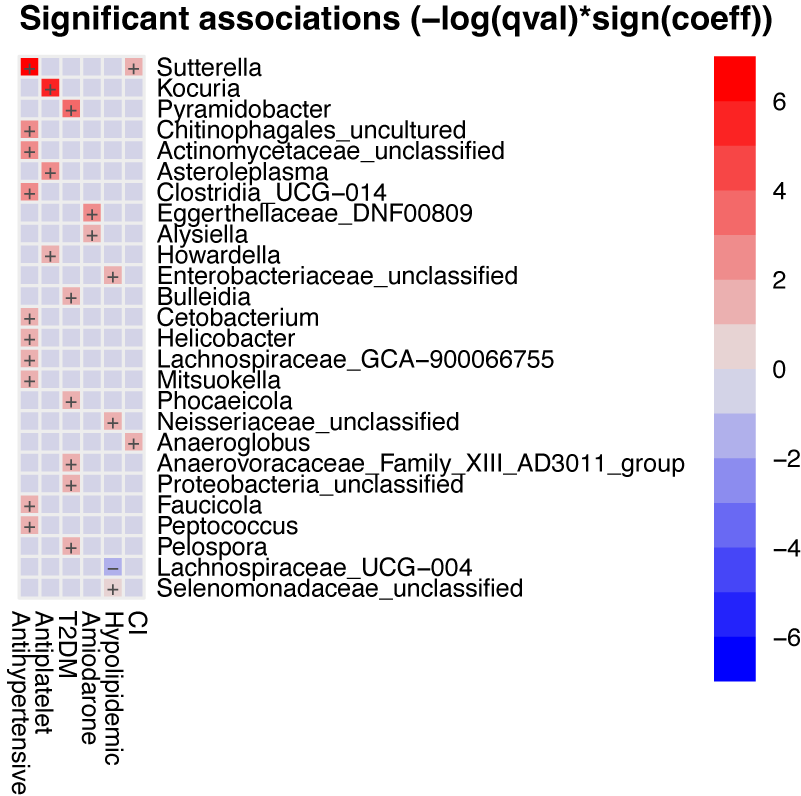

Supplement: SUPPLEMENTARY FIGURE S1 — Heatmap of tongue flora associated with medication use and medical history data at genus level. T2DM, type 2 diabetes mellitus; CI, cerebral infarction. [file Image_1.TIF]

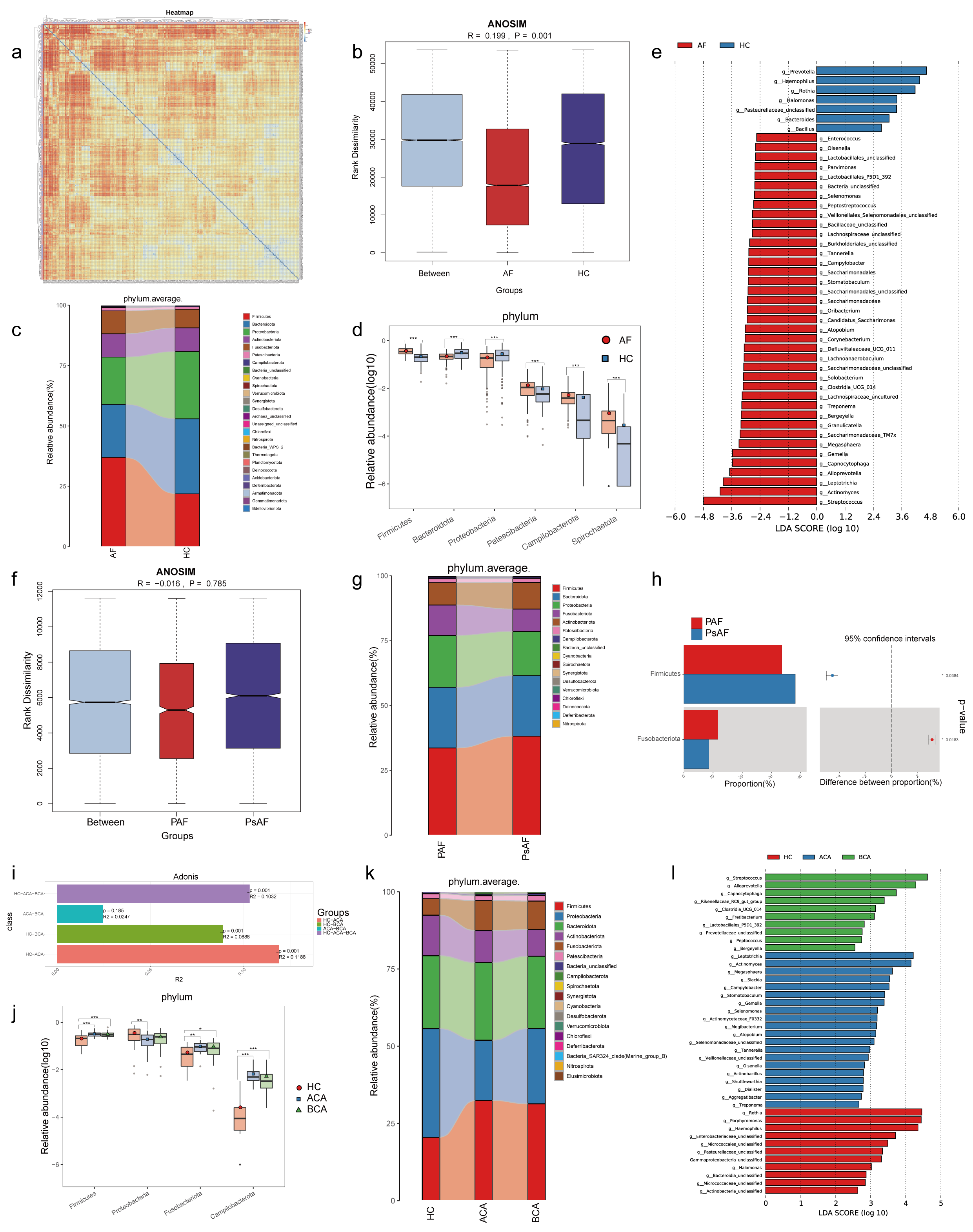

Supplement: SUPPLEMENTARY FIGURE S2 — (a) Heatmap based on Bray-Curtis distances reflect the degree of variation in species abundance distributions among samples. (b) Box plots of the results of ANOSIM’s analysis of similarity between AF and HC groups and comparisons of differences within and between groups. (c) Average compositions and relative abundance of the microbial community in AF and HC groups at the phylum level. (d) 6 phyla were remarkedly increased, while 2 phyla were remarkedly reduced in AF versus controls. (e) Linear discriminant analysis (LDA) effect size in oral microbiome between AF and HC groups at genus level (LDA > 2.5, p < 0.05). (f) Box plots of the results of ANOSIM’s analysis of similarity between PAF and psAF groups and comparisons of differences within and between groups. (g) Average compositions and relative abundance of the microbial community in PAF and psAF groups at the phylum level. (h) 1 phylum was remarkedly increased, while 1 phylum was remarkedly reduced in psAF versus PAF. (i) Beta diversity calculated by Adonis among ACA, BCA, and HC groups. (j) The differential microbes among ACA, BCA, and HC groups at the phylum level. (k) Average compositions and relative abundance of the microbial community in ACA, BCA, and HC groups at the phylum level. (l) LDA effect size in oral microbiome among ACA, BCA, and HC groups at genus level (LDA > 2.5, p < 0.05). AF, atrial fibrillation; HC, healthy control; PAF, paroxysmal AF; psAF, persistent AF; ACA, 6 months after catheter ablation; BCA, before catheter ablation. *p < 0.05, **p < 0.01, ***p < 0.001. [file Image_2.TIF]
