# Supplementary material for: The tongue coating microbiome is perturbed in atrial fibrillation and partly normalized after catheter ablation
Source: Front Microbiol. 2025 Apr 30;16:1508089. doi: 10.3389/fmicb.2025.1508089 (PMC12075123; doi:10.3389/fmicb.2025.1508089)
Supplement: Supplementary file 4 [file Data_Sheet_1.DOCX]

**Diagnostic, inclusion and exclusion criteria**

Diagnostic criteria are based on ‘Current knowledge and management of atrial fibrillation: consensus of Chinese experts 2021’^1^.

|  | Diagnostic criteria |
| --- | --- |
| AF | ECG documentation is required to establish the diagnosis of AF. A standard 12-lead ECG recording or a single-lead ECG tracing of >30s showing heart rhythm with no discernible repeating P waves and irregular RR intervals (when atrioventricular conduction is not impaired) is diagnostic of clinical AF. |
| PAF | AF that terminates spontaneously or with intervention within 7 days of onset. |
| psAF | AF that is continuously sustained beyond 7 days, including episodes terminated by cardioversion (drugs or electrical cardioversion) after ≥7 days. |

**Inclusion criteria**

1) Patients with a confirmed diagnosis of AF.

2) Sex is not limited.

3) Age 20 to 85 years.

4) The American Society of Anesthesiologists (ASA) Physical Status classification: Class I to III.

5) Voluntary acceptance of the study and signing of informed consent.

**Exclusion criteria**

1) Emergency surgery.

2) Preoperative pulmonary infection or uncontrolled acute lung disease or acute exacerbation of chronic obstructive pulmonary disease.

3) Combination of autoimmune disease, liver disease, renal disease, or malignant tumor.

4) Failure to awaken after surgery; perioperative death with automatic discharge.

5) Clinically definitive diagnosis of severe coagulation disorders.

6) Severe postoperative cerebral, hepatic, and renal insufficiency.

7) Serious infectious diseases, etc.

8) Incomplete clinical data.

9) Drug addiction or long-term use of psychotropic drugs; long-term use of steroids and hormonal drugs.

**Healthy volunteers**

**Inclusion criteria.**

1) Aged ≥18 years old.

2) Competent to provide informed consent (no mental illness or dementia).

3) Have lived in the same geographic area for the preceding 6 months.

4) All enrolled individuals had healthy oral tissues and gingiva (determined by a professional dentist).

**Exclusion criteria**

1) Individuals who received antibiotics and/or probiotics within 8 weeks before enrolment were also excluded.

2) Current pregnancy.

3) The use of laxatives or anti-diarrheal drugs in the last 3 months.

4) Have no underlying infectious, acute disease, or severe organ failure.

5) Hypertension, diabetes, obesity, metabolic syndrome, nonalcoholic fatty liver disease, coeliac disease and liver cirrhosis.

All enrolled individuals had healthy oral tissues and gingiva (determined by a professional dentist). Individuals who received antibiotics and/or probiotics within 8 weeks before enrollment were also excluded.

**Construction of POD**

We used the abundance profile of the optimal OTUs markers in the discovery set to perform fivefold cross-validation on a random forest model (R 3.4.1, randomForest 4.612 package). Then we acquired the cross-validation error curve through five trials of the fivefold cross-validation. We defined the point with the minimum cross-validation error as the cut-off point through the minimum error plus the standard deviation (SD).

The sets of OTU markers with the error less than the cut-off value were listed and the set with the smallest number of OTUs were defined as the optimal set. Then we used the optimal set of OTUs to calculate the probability of disease (POD) index in discovery cohort and validation cohort. The constructed models were evaluated using the receiver operating characteristic (ROC) curve (R 3.3.0, pROC package), and the ROC effect was showed using AUC.

1 Chinese Society of Pacing and Electrophysiology, Chinese Society of Arrhythmias, Atrial Fibrillation Center Union of China. Current knowledge and management of atrial fibrillation: consensus of Chinese experts 2021. *Chin J Cardiac Arrhyth* **26**, 15-88, doi:10.3760/cma.j.cn113859-20211224-00264 (2022) (China).
